# Surgery: a crucial ally for universal palliative care access

**DOI:** 10.1016/S2214-109X(23)00066-9

**Published:** 2023-05

**Authors:** William E Rosa, Juliet S Lumati, Olusegun I Alatise, Eve Namisango, Netsanet Bogale, Pedro E Pérez-Cruz, M R Rajagopal, Lesley Taylor, T Peter Kingham

**Affiliations:** Department of Psychiatry and Behavioral Sciences; Memorial Sloan Kettering Cancer Center, New York, NY 10017, USA; Division of Surgical Oncology, Department of Surgery, Johns Hopkins Medicine, Baltimore, MD, USA; Department of Surgery, College of Health Sciences, Obafemi Awolowo University, Ile Ife, Nigeria; African Research Group for Oncology, Ile Ife, Nigeria; African Palliative Care Association, Kampala, Uganda; College of Medicine and Health Sciences, Hawassa University, Hawassa, SNNPR, Ethiopia; Sección Medicina Paliativa, Facultad de Medicina, Pontificia Universidad Católica de Chile (UC-Chile), Santiago, Chile; Trivandrum Institute of Palliative Sciences, Thiruvananthapuram, Kerala, India; Pallium India, Trivandrum, India; Division of Breast Surgery, Department of Surgery, City of Hope Comprehensive Cancer Center, Duarte, CA, USA; Department of Surgery and Global Cancer Disparities Initiative; African Research Group for Oncology, Ile Ife, Nigeria

5 billion people worldwide do not have access to surgical and anaesthesia care,^1^ whereas an estimated 61 million people each year have serious health-related suffering amenable to palliative care.^2^ Although palliative care is a key component of universal health coverage,^3^ 64% of countries—mostly low-income and middle-income countries (LMICs)—have no or extremely poor access to palliative care.^2^ LMIC populations account for more than 80% of the global palliative care need, and although its integration throughout health systems is imperative to bridge global divides, people in LMICs disproportionately face death and disability from life-limiting illness and injury.^2,4^ The prevalence of decedents with health-related suffering will rise about 87% by 2060, most severely impacting LMICs and primarily driven by increased cancer deaths.^5^ Thus, there is great urgency to forge sustainable alliances between multidisciplinary surgical teams and palliative care specialists to address current and future unmet needs globally, but especially in LMICs.

Surgeons and multidisciplinary surgical teams are vital contributors to fully integrated palliative care services throughout the care continuum—from serious illness diagnosis to surgical intervention (whether curative or palliative), and during end-of-life care. These teams are often a unique point of contact between patients and health-care systems. Surgeons can make substantive contributions to palliative care provision through expert clinical decision making and technical skills, as well as person-centred and family-centred communication and building relationships to ensure goal-concordant services and optimal quality of life for the patient.^6^ Additionally, accessible and affordable pain management is an ethical priority of surgical and palliative care specialists. The poorest 50% of the global population live in countries with access to only 1% of the annually distributed morphine-equivalent opioid stock.^2^ Such inadequate access to evidence-based pain and symptom management is a substantial barrier for patients living in LMICs.^1^ These inequities are compounded by the paucity of formalised programmes for palliative care training in LMICs, with few exceptions.^1^ In some cases, surgeons are the only available health-care professionals treating patients with health-related suffering in LMICs given the shortages of a workforce specialised in palliative care.^4^ Thus, interdisciplinary approaches between surgery and palliative care are crucial to improving a streamlined, cross-team partnership with a range of health-care professionals, such as nurses, who comprise nearly 60% of the global health-care workforce and have the numeric power and clinical competency to effectively implement point-of-care and system-wide practice changes in palliative care access and delivery.^7^

There are ample opportunities to involve surgical care teams in providing palliative care services in LMICs, thereby alleviating the experience of suffering for patients, family caregivers, and communities. In Chile, almost half of the 727 362 surgeries done in 2021 were for patients with conditions requiring ambulatory surgical procedures and placed on waiting lists that often delay the relief of suffering.^8^ A palliative care approach in these circumstances would empower surgical teams to identify the underlying causes of distress and partner across specialties to recommend and deliver improved support. In robust, global academic–practice partnerships, such as in Ethiopia,^9^ there is a need for surgical–palliative care collaborations to strengthen the quality of service and overall care delivery, and to promote the reintegration of patients into the community after treatment interventions. For example, strategic surgical-palliative care interventions that emphasise early and ongoing symptom management and long-term, whole-person needs assessments could help to narrow the disparities in 2-year post-surgical survival rates in patients with breast cancer in urban (74%) and rural (46%) areas, which might be partly attributed to stigmas associated with the disease and treatments (eg, mastectomy).^9^ In India—with longstanding governmental and non-governmental palliative care provision^10^—multidisciplinary palliative care specialists can provide general and targeted palliative care training that can be adapted by surgical teams to meet their unique service and patient needs. Additionally, palliative care specialists and surgeons in these settings can merge resources to advocate for balanced, controlled, and essential access to medicines (eg, opioids for moderate to severe pain and breathlessness at end of life) while identifying regulatory pathways needed to mitigate opioid misuse and diversion.

There should be international and multi-stakeholder commitment to embed competencies in primary palliative care throughout surgical curricula and global health research endeavours. Such skills will equip surgical teams to foster relationship-based care with patients, caregivers, and communities; optimise pain and symptom management; elicit patients’ core values; provide psychosocial screening and care; and participate in iterative advance care planning in the face of unpredictable serious illness and perioperative courses. Increased investments in palliative care will also empower investigators to ensure research is designed and implemented from a community-based and people-centred stance.

Several recommendations for surgical teams and palliative care specialists are provided (panel). We recognise the substantive resource constraints facing surgical teams and do not suggest formalised palliative care delivery as another task shifting expectation. Rather, our suggestions are about task integration. Ultimately, learning to integrate palliative care principles in everyday surgical practice will improve perioperative quality, person-centredness, and physical and psychosocial outcomes through holistic assessment and intervention. Data are needed to understand the perspectives of surgical teams on how best to increase the knowledge of palliative care in surgical training, practice, and research.

Arguably, all surgical teams should be palliative care oriented,^11^ capable of grasping patients’ narratives, values, preferences, and goals, while using empathic communication skills to measurably inform outcomes. The capacity of surgical teams to build on palliative care practices prepares them to facilitate encounters that transcend disease-focused or transactional interventions. Palliative care provision at the surgical–generalist level will move us towards care models that honour patients’ personhood and wellbeing while enabling health systems in LMICs to make universal health coverage (inclusive of palliative care) a societal reality.

## *Panel:* Recommendations for surgical teams and palliative care specialists to increase palliative care access

### Surgical teams

To increase palliative care access, surgical teams should:

- Integrate generalist-level palliative care skills as a fundamental surgical competency in basic and continuing education programmes
- Seek advanced training in evidence-based pain and symptom management for acute and chronic malignant and non-malignant health conditions
- Provide education in analgesic and pain management for multidisciplinary health-care professionals to alleviate preventable suffering, minimise perioperative pain, and reduce postoperative complications
- Engage in discussions about goals of care and promote shared decision making at all stages of surgical care (eg, curative *vs* palliative surgical interventions, risks of surgical procedures *vs* benefits of comfort-focused, non-surgical approach)
- Prioritise multidisciplinary roles in clinician–patient and caregiver communication to elicit physical, psychosocial, spiritual, cultural, ethical, and other needs related to life-limiting illness and injury

### Palliative care specialists

To increase palliative care access in collaboration with surgical partners, palliative care specialists should:

- Offer palliative care services, consultation, and education to surgical collaborators at all levels of care and in all settings
- Establish local, regional, national, and global partnerships between palliative care and surgical organisations and professional associations to identify joint clinical and research agendas
- Invite surgeons and members of the surgical team to contribute their expertise and insights during patient and family meetings, conversations about goals of care, and while mediating ethical dilemmas to role model partnership and inclusion of all clinicians and surgical team experts
- Identify institution-specific and population-specific research endeavours that will be beneficial to the communities being served, as well as to surgical and palliative care teams
- Redesign palliative care services to include surgical team member roles to leverage surgical skillsets, increase clinician knowledge and care access, and facilitate cross-team and cross-system collaboration

## References

[1] Meara JG, Leather AJ, Hagander L, Global Surgery 2030: evidence and solutions for achieving health, welfare, and economic development. Lancet 2015; 386: 569–624. 25924834 10.1016/S0140-6736(15)60160-X

[2] Knaul FM, Farmer PE, Krakauer EL, Alleviating the access abyss in palliative care and pain relief—an imperative of universal health coverage: the *Lancet* Commission report. Lancet 2018; 391: 1391–454. 29032993 10.1016/S0140-6736(17)32513-8

[3] World Health Organization. Strengthening of palliative care as a component of integrated treatment within the continuum of care. 2014. https://apps.who.int/gb/ebwha/pdf_files/EB134/B134_R7-en.pdf(accessed Feb 22, 2023). 10.3109/15360288.2014.911801 24779434

[4] Connor S, Morris C, Jaramillo E, Global atlas of palliative care, 2nd edn. 2020. http://www.thewhpca.org/resources/global-atlas-on-end-of-life-care (accessed Feb 22, 2023).

[5] Sleeman KE, de Brito M, Etkind S, The escalating global burden of serious health-related suffering: projections to 2060 by world regions, age groups, and health conditions. Lancet Glob Health 2019; 7: e883–92. 31129125 10.1016/S2214-109X(19)30172-X PMC6560023

[6] Miner TJ, Cohen J, Charpentier K, McPhillips J, Marvell L, Cioffi WG. The palliative triangle: improved patient selection and outcomes associated with palliative operations. Arch Surg 2011; 146: 517–22. 21576604 10.1001/archsurg.2011.92

[7] WHO. State of the world’s nursing 2020: investing in education, jobs, and leadership. 2020. (accessed Feb 22, 2023).

[8] Subsecretaría de Redes Asistenciales, Ministerio de Salud de Chile. Informe de Situación Financiera, Asistencial y de Dotación de Personas y Licencias Médicas (Cuatro Trimestre). 2021. https://www.minsal.cl/wp-content/uploads/2021/05/ORD-851-DIGERA-Glosa-04.pdf (accessed Feb 22, 2023).

[9] Taylor L, Harris C, Abebe T, Addissie A, Assefa M, Kantelhardt EJ. A decade of strengthening breast oncology in Ethiopia. Lancet Oncol 2021; 22: 1219–20.

[10] Lijimol AS, Krishnan A, Rajagopal MR, Gopal BK, Booth CM. Improving access and quality of palliative care in Kerala: a cross-sectional study of providers in routine practice. Indian J Palliat Care 2020; 26: 500–05. 33623312 10.4103/IJPC.IJPC_17_20 PMC7888431

[11] Katlic MR. All surgeons should be palliative care surgeons. JAMA Surg 2022; 157: 1132–33. 36260364 10.1001/jamasurg.2022.4725

